# γ-PGA-Rich Chungkookjang, Short-Term Fermented Soybeans: Prevents Memory Impairment by Modulating Brain Insulin Sensitivity, Neuro-Inflammation, and the Gut–Microbiome–Brain Axis

**DOI:** 10.3390/foods10020221

**Published:** 2021-01-21

**Authors:** Do-Youn Jeong, Myeong Seon Ryu, Hee-Jong Yang, Sunmin Park

**Affiliations:** 1 Department of Research and Development, Sunchang Research Center for Fermentation Microbes, Sunchang-Gun 56048, Korea; jdy2534@korea.kr (D.-Y.J.); rms6223@naver.com (M.S.R.); godfiltss@naver.com (H.-J.Y.); 2 Department of Food and Nutrition, Obesity/Diabetes Research Center, Hoseo University, 165 Sechul-Ri, BaeBang-Yup, Asan-Si 31499, ChungNam-Do, Korea

**Keywords:** chungkookjang, cheonggukjang, soybean, *Bacillus*, glucose metabolism, memory function, gut microbiota

## Abstract

Fermented soybean paste is an indigenous food for use in cooking in East and Southeast Asia. Korea developed and used its traditional fermented foods two thousand years ago. Chungkookjang has unique characteristics such as short-term fermentation (24–72 h) without salt, and fermentation mostly with *Bacilli*. Traditionally fermented chungkookjang (TFC) is whole cooked soybeans that are fermented predominantly by *Bacillus* species. However, *Bacillus* species are different in the environment according to the regions and seasons due to the specific bacteria. *Bacillus* species differently contribute to the bioactive components of chungkookjang, resulting in different functionalities. In this review, we evaluated the production process of poly-γ-glutamic acid (γ-PGA)-rich chungkookjang fermented with specific *Bacillus* species and their effects on memory function through the modulation of brain insulin resistance, neuroinflammation, and the gut–microbiome–brain axis. *Bacillus* species were isolated from the TFC made in Sunchang, Korea, and they included *Bacillus (B.) subtilis*, *B. licheniformis,* and *B. amyloliquefaciens*. Chungkookjang contains isoflavone aglycans, peptides, dietary fiber, γ-PGA, and *Bacillus* species. Chungkookjangs made with *B. licheniformis* and *B. amyloliquefaciens* have higher contents of γ-PGA, and they are more effective for improving glucose metabolism and memory function. Chungkookjang has better efficacy for reducing inflammation and oxidative stress than other fermented soy foods. Insulin sensitivity is improved, not only in systemic organs such as the liver and adipose tissues, but also in the brain. Chungkookjang intake prevents and alleviates memory impairment induced by Alzheimer’s disease and cerebral ischemia. This review suggests that the intake of chungkookjang (20–30 g/day) rich in γ-PGA acts as a synbiotic in humans and promotes memory function by suppressing brain insulin resistance and neuroinflammation and by modulating the gut–microbiome–brain axis.

## 1. Introduction

Soybeans have been consumed as a primary source of protein in unfermented and fermented forms in East Asian countries, where rice is the main staple. Lysine and methionine are limited amino acids in rice and soybeans, respectively. Rice and soybeans are complementary protein sources and together make a complete protein source. Although dried soybeans have long storage durations, they need to be soaked and boiled before eating. Boiled soybeans were initially fermented to preserve them for longer periods. According to the fermentation conditions, the degradation of soybean components is varied and different types of fermented soybeans are produced. In Korea, soybeans are fermented with and without salt. The salt-added soybeans are fermented for several months to several years, while soybeans without salt have a short-term fermentation of 2–3 days. In Korea, short-term fermented soybean without salt is called chungkookjang (some studies spell it as cheonggukjang), while long-term fermented soybeans with salt are doenjang and kochujang with added red peppers. According to short- and long-long term fermentation, the microorganisms that ferment soybeans are different. The primary microorganisms are *Aspergillus* and *Bacillus* to make meju, the component of doenjang and kochujang [1]. They are fermented for more than six months, and salt concentrations influence the type and relative abundance of microorganisms, and the bioactive components are varied [2]. Their quality controls are more difficult. However, chungkookjang is made by 2–3 day fermentation, and the primary microorganisms are *Bacillus* species. The bacteria for chungkookjang can be easier to control than doenjang. Small differences in the bacteria can result in different bioactive compounds and flavors that contribute to their functionality for health and palatability. Furthermore, the microorganisms in the fermented soybeans can act as probiotics, and they also contain prebiotics such as dietary fibers and poly-γ-glutamic acid (γ-PGA) [3]. As a result, fermented soybeans act as synbiotics.

The fermented soybeans have been reported to have more beneficial functions than unfermented soybeans for type 2 diabetes when they are optimally made [4,5,6,7,8]. Soybeans contain proteins, isoflavone glycosides, soluble dietary fiber, and fats, and they are beneficial for glucose and lipid metabolism [9,10,11]. Traditionally made chungkookjang is known for better alleviating energy and glucose dysregulation, memory impairment, and immunity compared with unfermented soybeans in animal and human studies [4,6,7]. The differences are associated with bacterial-driven changes of soybean components and bacteria that modify their absorption in the gastrointestinal tracts and gut microbiota [4,7]. In this review, we explain the production process of γ-PGA-rich chungkookjang fermented with individual *Bacillus* species. The efficacy of the inoculated chungkookjang is also evaluated for improving memory function by modulating brain insulin resistance, neuroinflammation, and the gut–microbiome–brain axis. 

## 2. Chungkookjang Processing and Bioactive Components 

### 2.1. Bioactive Components According to the Methods of Chungkookjang Processing 

**Figure 1:**
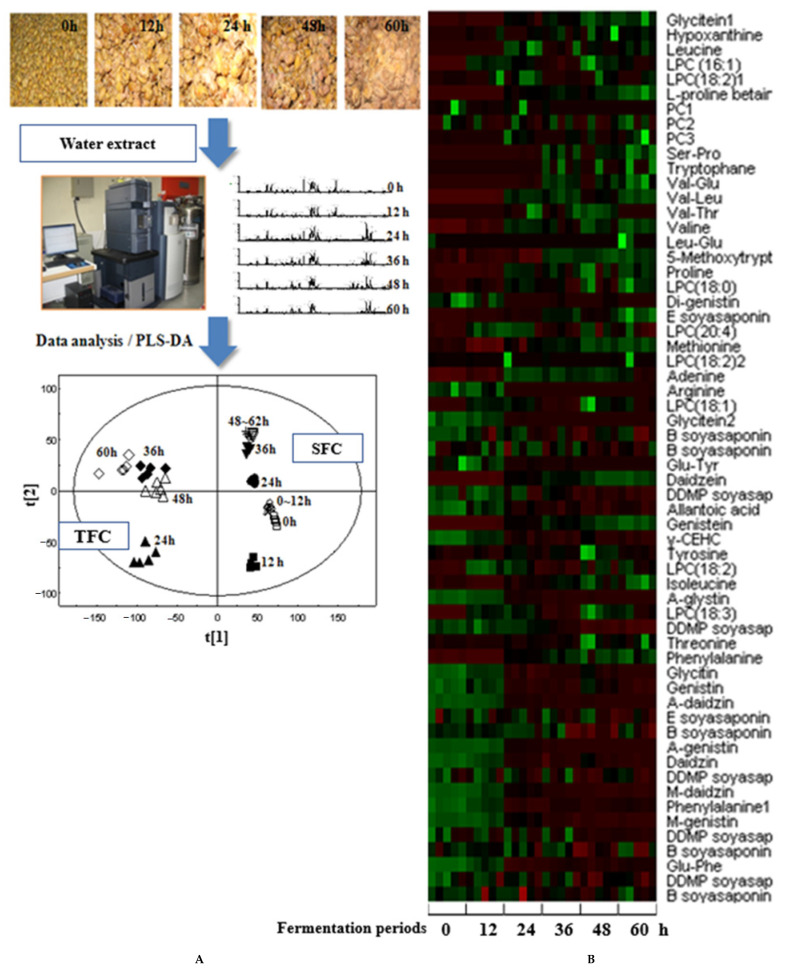
The metabolomic analysis of traditionally made chungkookjang (TFC) and chungkookjang fermented with *Bacillus licheniformis* (SFC). (**A**) Soybeans fermented for 0 to 60 h with TFC and SFC, and the separation of bioactive components in the unfermented and fermented soybeans by partial least squares-discriminant analysis (PLS-DA) [7]. (**B**) Heatmap of bioactive components of soybeans fermented with *Bacillus licheniformis* for 0, 12, 24, 36, 48, and 60 h [7]. LPC, lysophosphatidylcholine; PC, phosphatidyl choline; Ser, serine; Pro, proline, Glu, glutamic acid; Leu, leucine, Thr, threonine; Tyr, tyrosine; Val, valine; DDMP, 2,3-Dihydro-2,5-dihydroxy-6-methyl-4H-pyran-4-one; Phe, phenylalanine.

When preparing chungkookjang, dried soybeans are sorted, washed, and soaked in water for 12 h at 15 °C and then boiled for 4 h at 100 °C. The cooked soybeans are cooled to 40 °C and fermented with rice straw in a fermentation chamber at 30 °C for 24–72 h to make chungkookjang by traditional methods (Figure 1A) [12]. Traditionally made chungkookjang contains several types of *Bacilli,* mainly *B. subtilis, B. licheniformis*, and *B. amyloliquefaciens*. The types of *Bacilli* in chungkookjang depend on the ambient bacteria present in the environment [7]. In the last decade, research has been conducted to explore the optimal *Bacillus* for chungkookjang to have various biological functionalities in research on animals and humans [7,13]. According to the fermentation conditions such as fermentation areas and periods, the bioactive components in chungkookjang are different. Accordingly, chungkookjang has different functionalities, as shown in the metabolomic analysis of fermented soybean extracts [7]. For example, soybeans fermented for 0 to 60 h by traditional methods and chungkookjang fermented with inoculated *Bacillus* produce different bioactive components from the same unfermented soybeans as revealed by partial least squares-discriminant analysis (PLS-DA) [7]. The optimal fermentation time is 48 h in most cases (Figure 1A) [7]. The components and functionalities of traditionally made chungkookjang are different due to the fermentation with different bacteria (mainly *B. subtilis, B. licheniformis*, and *B. amyloliquefaciens*) found in different environments. Chungkookjang fermented with *B. licheniformis* also produces different types and amounts of peptides (Val-Glu, Val-leu, Val-Thr, Leu-Glu), amino acids (tyrosine, arginine, threonine), γ-PGA, phospholipids, isoflavone aglycans, and soyasaponins during the fermentation periods, as measured in the metabolomic analysis (Figure 1B) [7]. Traditionally made chungkookjang and chungkookjang inoculated with specific *Bacillus* species exhibit different amounts of bioactive components (Figure 1B). The different types of chungkookjang demonstrate unique functionalities. Chungkookjang containing high γ-PGA has better efficacy for improving glucose metabolism and neuronal cell survival [5]. Fermenting with specific *Bacillus* species to change bioactive components, including the generation of higher γ-PGA, may enhance the functionality of chungkookjang [7]. 

### 2.2. Functionality of Chungkookjang According to Bacillus Species

Chungkookjang traditionally made in Sunchang, Chonbuk-Do, Korea, has been reported to promote insulin sensitivity and subsequently to protect against type 2 diabetes and Alzheimer’s disease progression [5,14,15]. Subjects who consumed traditionally made chungkookjang (26 g/day) exhibited a decrease in apolipoprotein B compared to those who consumed a placebo (*p* < 0.05) in a 12-week randomized clinical trial [16]. The visceral fat mass measured by computerized tomography scans tended to decrease in the subjects who consumed chungkookjang, compared to those who had the placebo. Therefore, chungkookjang is effective for protecting the health of humans. Traditionally made chungkookjang from Sunchang, Korea, is standardized chungkookjang with consistently high efficacy. The method for standardization of chungkookjang production by inoculating specific *Bacillus* species involves the inoculation with specific bacteria isolated from traditionally fermented chungkookjang from Sunchang. The major bacteria isolated from chungkookjang are *Bacillus (B.) subtilis*, *B. amyloliquefaciens*, and *B. licheniformis* [12,14]. Each *Bacillus* spp. has various strains of the same bacterial species, and they have bioactive functions with different efficacies in vitro and in vivo studies [12.14]. Therefore, the types of *Bacillus* species and strains play a key role in the functionalities of chungkookjang. The daily intake (20–30 g) of chungkookjang fermented with *B. amyloliquefaciens* and *B. licheniformis* is beneficial for glucose and lipid metabolism in humans. 

## 3. Types and Characteristics of *Bacillus* Species Isolated from Traditionally Made Chungkookjang

### 3.1. Toxic Bacillus Species Potentially Existing in Chungkookjang

*Bacillus* species are ubiquitous, and they are rod-shaped, endospore-forming aerobic, or facultatively anaerobic bacteria [17]. *Bacilli* exhibit a wide range of physiologic abilities, but some species of *Bacillus* act as pathogenic bacteria. They may produce either tissue-damaging toxins or metabolites such as penicillinase to degrade antibiotics [17]. The major pathogenic *Bacillus* species in humans is *B. cereus*, and it acts as an agent of food poisoning to produce tissue-damaging toxins [17]. *B. cereus* contamination needs to be determined in the products fermented with *Bacillus* species such as chungkookjang. Chungkookjang made with *B. amyloliquefaciens* and *B. subtilis* does not contain *B. cereus* (Table 1)*. B. licheniformis* is also recognized as infectious and toxic bacteria followed by *B. cereus* [17]. However, the symptoms of food poisoning caused by *B. licheniformis* are not well characterized [18]. By contrast, *B. licheniformis* SCK 121057 suppresses *B. cereus-*producing toxins, and *B. licheniformis* strains 141 have beneficial activities for promoting life span [19] and improving hyperglycemia in animal studies [20,21]. The policy for *B. licheniformis* is different in different countries. *B. licheniformis* is not registered for foods in Korea while it is available in a dried form as a probiotic in China [22,23]. *B. licheniformis* needs to be studied more to determine its toxicity for use as foods since some strains of *B. licheniformis* have probiotic activities, and chungkookjang fermented with *B. licheniformis* improves the bioactivities in animal studies [24]. 

### 3.2. Characteristics of Bacillus Species Isolated from Chungkookjang

The type of *Bacillus* in chungkookjang influences the flavor of chungkookjang, mostly by modulating the fatty acids produced during fermentation [25] that play a crucial role in the flavor and aroma, which determine consumer acceptance. Chungkookjang made with *B. amyloliquefaciens* has a less unacceptable odor and greater consumer acceptance (Table 1) [24,26,27]. The dominant *Bacillus* species of chungkookjang is *B. subtilis*, but *B. subtilis*, *B. amyloliquefaciens,* and *B. licheniformis* and their different strains are all present in traditionally made chungkookjang. *B. subtilis* and *B. amyloliquefaciens* have been isolated and characterized at the Sunchang Research Center for Fermentation Microbes (Sunchang, Korea) [24,27]. The isolated *Bacillus* species and strains have been studied for different bioactivities. Chungkookjang fermented with *B. amyloliquefaciens* and *B. subtilis* contains 1.5 × 10^9^ – 2.2 × 10^10^ bacteria, but does not contain any *B. cereus* (Table 1). Surprisingly, unfermented soybeans contained 5.0 × 10^3^ bacteria after 48 h incubation without *Bacillus* inoculation, and they were mostly *B. cereus*. Therefore, boiled soybeans can be easily contaminated with *B. cereus* during incubation, but the predominant *Bacillus* prohibits the growth of *B. cereus*. *B. amyloliquefaciens* SCGB 1, SRCM 100730, and SRCM 100731, and *B. subtilis* SCGB 574 produce high contents of γ-PGA (Table 1), and chungkookjang made with these *Bacilli* have a flavor that is acceptable to people. Chungkookjang made with *B. amyloliquefaciens* SCGB 1 and SRCM 100730 and *B. subtilis* SCGB 574 has high in vitro activities of protease, cellulase, and amylase [24,28]. Furthermore, the chungkookjang made with *B. amyloliquefaciens* SRCM 100730, and SRCM 100731 had the highest thrombolytic activity in in vitro studies [24,28], suggesting potential health benefits (Table 1). 

### 3.3. Production of γ-PGA in Chungkookjang According to Bacillus Species

Chungkookjang, fermented with *B. licheniformis*, produces higher contents of γ-PGA during soybean fermentation, and the fermented soybeans improve cognitive dysfunction and hyperglycemia by promoting insulin sensitivity and glucose-stimulated insulin secretion [6,7,20,29]. *B. licheniformis* is also used as a probiotic in China [22,23]. *B. licheniformis, B. amyloliquefaciens,* and *B. subtilis* produce high γ-PGA (a polymer of glutamate) and levan (a polymer of fructose with 2,6-beta glycosidic linkages) (Table 1). Levan and γ-PGA have anti-oxidant, anti-fungal, and anti-microbial activities and improve immunity by modulating gut microbiota in animal studies [30,31,32]. These studies suggested that *B. licheniformis* has the potential to contribute probiotic and/or synbiotic benefits to the soybeans. However, *B. licheniformis* is not registered as a food ingredient in Korea, and it cannot be used for food production in Korea. Further studies need to confirm that *B. licheniformis* does not have toxicity and can be considered a probiotic.

### 3.4. Characteristics of B. subtilis and B. amyloliquefaciens as Probiotics

Chungkookjang fermented with *B. subtilis* and *B. amyloliquefaciens* has been studied for its microbial bioactivity [5,33]. Different strains of *B. amyloliquefaciens* and *B. subtilis* are isolated from traditionally made chungkookjang from Sunchang, Korea [12], and their probiotic properties have been studied in vivo. Probiotics are defined as live microorganisms that exist in the gut upon ingestion to exert health benefits to the host. The microorganisms need to have acid-resistance, bile resistance, anti-bacterial activity, no hemolytic activity, and exhibit bioamine degradation; some *B. amyloliquefaciens* and *B. subtilis* have these probiotic properties (Table 2) [28]. In our preliminary and previous in vitro studies [24,28], *B. amyloliquefaciens* SRCM 100731 had a greater survival rate at pH 2.0 than *B. amyloliquefaciens* SCGB 1 and SRCM 100730 and *B. subtilis* SCGB 574, although *B. amyloliquefaciens* SRCM 100731 showed only a 6.91% survival rate (Table 2). They all had some bile salt resistance in 0.3% oxagall. *B. amyloliquefaciens* SCGB 1, SRCM 100730, SRCM 100731, and *B. subtilis* SCGB 574 had survival rates in the range of 19.8–35.04%. In 0.6% oxagall, only *B. amyloliquefaciens* SRCM 100730 and SRCM 100731 showed bile salt resistance (Table 2). These bacillus bacteria also showed anti-microbial activity against *B. cereus* and *Staphylococcus aureus* [24,28]. Chungkookjang fermented with *B. amyloliquefaciens* SRCM 100730 and SCGB 1 also exhibited anti-inflammatory activities by the inhibition of the expression of mRNAs of inducible nitric oxide synthase, tumor necrosis factor-α (TNF-α), and IL-6 in lipopolysaccharide-stimulated RAW 264.7 macrophages [27,34]. These *Bacillus* bacteria also produced anti-bacterial components, including surfactin, iturin A, and bacillomycin D [27,28]. These characteristics suggest that *B. amyloliquefaciens* SCGB 1, SRCM 100730, and SRCM 100731 can be used as probiotics.

## 4. Components of Soybeans that Function as Prebiotics to Reduce the Risk of Metabolic Diseases 

### 4.1. Dietary Fiber 

Soybeans contain 16% dietary fiber, 21% protein, 2.1% carbohydrates, and 1.2% fats. They also contain β-D-glucoside isoflavones (daidzin, glycitin, and genistin) and their aglycones (daidzein, glycitein, and genistein) [35]. Soybeans are good sources of protein for people who consume grains as a staple food since soybeans and grains have different limited amino acids, and they can be complementary for protein quality [36]. Moreover, soybeans are rich in soluble dietary fiber, including pectin and hemicellulose, which act as prebiotics in the gut. In a recent meta-analysis of prospective studies with soybean intake and type 2 diabetes [37], the intake of tofu, soy proteins, and isoflavonoids had an inverse association with the incidence of type 2 diabetes in humans, and the inverse associations were significantly linear with dose-responsiveness (*p* < 0.05). However, there was no significant association of type 2 diabetes with the consumption of total legumes, total soy, and soy milk [37]. The results suggest that the intake of soy protein and isoflavonoids has potential benefits to reduce the risk of type 2 diabetes. Moreover, soybean intake provides not only proteins and isoflavonoids but also dietary fiber, vitamins, and minerals [36]. Pectin, the predominant soybean dietary fiber, possesses a high water-holding ability (5.26 g water per g dietary fiber) and oil-holding capacity (4.83 g oil per g dietary fiber) [38]. In an in vitro study, soybean dietary fiber had high cholesterol-binding affinity, bile acid-binding activity, and glucose-binding ability [38]. This indicates that soy dietary fiber suppresses glucose and cholesterol absorption in the gut, and it modulates gut microbiota composition and contents. Soy milk increases the ratio of *Firmicutes* and *Bacteriodetes* due to an increase in *Lactobacillus* species, and soy milk with fiber greatly decreases the ratio of *Allobaculum* and *Parabacteroides* species, related to inflammation in rodents [39]. Soy dietary fiber intake exhibits a positive association with the relative abundance of *Lactobacillus, Ruminococcus, Bifidobacteriales*, and *Flavonifractor* at the genus level, which is linked to suppressing toll-like receptor (TLR)-4 → NF-kB signaling pathways in laboratory animals [40,41]. Gut microbiota uses soy dietary fiber as an energy source since it is a soluble dietary fiber. The components in chungkookjang promote its utilization in the gastrointestinal tracts. Accordingly, chungkookjang can be considered a prebiotic food. Therefore, it is not only soy protein but also soy dietary fiber that can mitigate the risk of metabolic diseases, and further studies need to be conducted. 

### 4.2. γ-PGA

Fermentation of soybeans changes the compositions of peptides, amino acids, dietary fiber, γ-PGA, and isoflavonoids according to the fermentation conditions, including the types of bacteria and fermentation duration [7,42]. Dietary fibers are degraded during fermentation, and the smaller fibers are microbiota-accessible. During the short-term fermentation of soybeans with *Bacillus*, the amounts of γ-PGA produced vary with different *Bacillus* species [43]. γ-PGA is composed of 5000–10.000 units of L-glutamate, D-glutamate, or both residues polymerized by γ-amide linkages [43]. It is a biocompatible and biodegradable polypeptide, and it has wide-ranging applications in foods, cosmetics, medicine, and agriculture [44]. *B. licheniformis* produces γ-PGA from glutamate, and the low intracellular glutamate concentration is the limiting factor for γ-PGA production. *B. amyloliquefaciens* LL3 also enhances γ-PGA production in vitro [44]. It is mainly used as a biofilm or hydrogel to encapsulate nanomaterials, including chemotherapeutic drugs for delivery into the body [45]. It has a short-term therapeutic activity in some infections without increasing natural killer cell activity and the major histocompatibility complex class II CD8 and CD56 count [46,47]. The γ-PGA property of coating bioactive components, including isoflavonoids and minerals, enhances their absorption to improve their bioactivities [48]. γ-PGA increases calcium solubility by chelation, and the chelated calcium ion exhibits enhanced absorption in the small intestines of the laboratory animals [49]. γ-PGA may also coat isoflavones and soyasaponins in chungkookjang to promote their utilization, including absorption from the gastrointestinal tract after consumption. Therefore, the higher contents of γ-PGA in chungkookjang, the greater the bioactivity of the soybeans.

### 4.3. Fermented Soybeans as Prebiotics

γ-PGA itself has bioactive properties: it stimulates and improves immune activity potentially by modulating gut microbiota and also suppresses pathogen growth [50]. In vivo and in vitro studies have demonstrated that γ-PGA acts as an enhancer of glucose tolerance in animal feeding studies and 3T3-L1 cell culture studies [51,52]. These results indicate that γ-PGA acts as a prebiotic in rodents and humans since γ-PGA is composed of the γ-linkage of glutamate residues, which are resistant to proteases in the gastrointestinal tracts in humans and animals [3]. Oral administration of γ-PGA increased the abundance of *Lactobacillales* while reducing the abundance of *Clostridiales* in the gut of rodents [3]. In KK type 2 diabetic mice, the 28-day intake of a diet containing 0.5% γ-PGA elevated *Lactobacillus* counts and tended to increase *Prevotella* counts and reduce visceral fat mass, compared to the placebo control group [53]. γ-PGA can be used as an energy source for gut microbiota, and it can be considered a prebiotic. As a result, chungkookjang contains dietary fibers and γ-PGA as prebiotics [3,24], and it also includes probiotic *Bacillus* species. Thus, chungkookjang can act as a synbiotic food.

## 5. Protection against Memory Impairment by Chungkookjang by Optimizing Systemic and Brain Glucose Metabolism and Suppressing Neuroinflammation

### 5.1. Chungkookjang Effect on Memory Impairment

Cerebral infarction and amyloid-β accumulation induce memory impairment, and chungkookjang intake has neuroprotective activities. Cerebral infarction results in neural cell death in the process of reduced blood flow, increased oxidative stress and inflammation, and elevated brain insulin resistance in animal studies [35,54]. Post-stroke hyperglycemia is associated with increased insulin resistance and β-cell death after ischemic stroke, and it exacerbates brain damage by increasing oxidative stress and inflammation in ischemia-induced gerbils. The intake of chungkookjang made with *B. amyloliquefaciens* and *B. licheniformis* (4.5% diet) increases blood flow measured by Doppler and reduces the concentrations of inflammatory cytokines, TNF-α, and interleukin (IL)-1β in gerbils with induced cerebral infarction [35,42]. It also prevents post-stroke hyperglycemia and improves brain insulin sensitivity. Chungkookjang was reported to increase the mRNA expression of brain-derived neurotrophic factor (BDNF) and ciliary neurotrophic factor (CNTF) in the hippocampus [35,42]. The consequent prevention of neuronal cell death and the increase in neuronal cell survival resulted in better post-stroke outcomes and memory function in gerbils [35,42]. In nerve growth factor (NGF)-induced PC12 cells, chungkookjang extracts prevented cell death by amyloid-β and potentiated CNTF and BDNF expression [5]. Therefore, chungkookjang intake has a beneficial effect on brain cell survival and brain insulin sensitivity in the ischemic and amyloid-β accumulation states.

### 5.2. Chungkookjang Effects on Systemic and Brain Glucose Metabolism

The impairment of systemic glucose metabolism is related to increased insulin resistance and insufficient insulin secretion. Chungkookjang traditionally made and fermented with *B. licheniformis* and *B. amyloliquefaciens* SRCM 100730 and 100731 has high γ-PGA contents. These types of chungkookjang enhance insulin sensitivity in adipocytes and the liver by stimulating peroxisome proliferator-activated receptor (PPAR)-γ activity and potentiating glucose-stimulated insulin secretion and pancreatic β-cell mass in rodents [4,5,6,14,55,56]. These results suggest that γ-PGA-rich chungkookjang enhances systemic glucose metabolism. Aging, obesity, inflammation, and oxidative stress induce systemic insulin resistance that contributes to brain insulin resistance [56]. Chungkookjang is also reported to reduce inflammation by suppressing NF-κB-dependent inducible nitric oxide synthase and cytokine production induced by TLR ligands in RAW264.7 cells [57]. The proinflammatory cytokines, including TNF-α and IL-1β, increase insulin resistance, which caused a concomitant stimulation of insulin release from the pancreatic β-cells regardless of serum glucose concentrations in an animal study [58]. The sustained increases in concentrations of proinflammatory cytokines induce hyperglycemia, which impairs brain glucose metabolism. Chungkookjang intake decreases proinflammatory cytokines indirectly to reduce brain insulin resistance. Therefore, chungkookjang intake has the potential to alleviate brain insulin resistance due to aging, high-fat intake, oxidative stress, and inflammation.

Brain insulin resistance is shown to a disturbance of brain insulin signaling in experimental animals, which is represented by the attenuation of insulin receptor substrate-2 (IRS2) → phosphoinositide 3-kinase → phosphorylated Akt → phosphorylated glycogen synthase kinase-3β (GSK-3β) [59]. Brain insulin resistance decreases glucose utilization of the brain, and it is linked to the low cerebral metabolic rate of glucose that occurs in people with cognitive decline and dementia [60,61]. Furthermore, systemic and brain glucose metabolism has demonstrated a linearly positive relationship in the healthy elderly [62]. Type 2 diabetes accelerates neurodegeneration by stimulating tau phosphorylation to increase amyloid-β deposition in the hippocampus of Alzheimer’s disease patients [56]. Hippocampal amyloid-β accumulation disturbs systemic glucose homeostasis by increasing hepatic insulin resistance and decreasing β-cell mass in rodents [59]. These results suggest that a low cerebral glucose metabolic rate is linked to higher cerebral glucose concentrations and reduced brain insulin sensitivity, contributing to Alzheimer’s disease pathologies in humans [59]. The reduction of IRS2 gene expression contributes to the attenuation of the brain insulin signaling pathway. It results in memory impairment and hyperglycemia.

### 5.3. Chungkookjang Effects on Systemic and Neuroinflammation and Neuronal Survival

**Figure 2:**
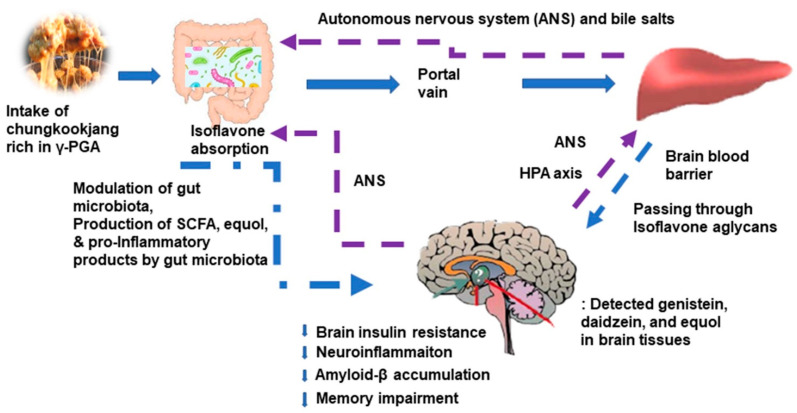
Potential action mechanism of chungkookjang in glucose metabolism and memory function. ANS, autonomous nervous system; HPA, hypothalamus–pituitary–adrenal; SCFA, short-chain fatty acids. Chungkookjang components directly influence glucose metabolism in the liver and brain, and they also indirectly affect them through the gut–microbiome–liver–brain axis. The improvement of glucose metabolism in the hippocampus reduces amyloid-b deposition, which decreases memory impairment. Thus, chungkookjang intake (about 20–30 g/day) protects against and partially alleviates type 2 diabetes, Alzheimer’s disease, and post-stroke symptoms.

In addition to brain insulin resistance, mitochondrial dysfunction and neuroinflammation elevate reactive oxygen species that activate β-secretase and γ-secretase. These enzymes produce amyloid-β from amyloid precursor proteins in mice and nonhuman primates [62]. Figure 2 suggests the possible mechanism of chungkookjang in glucose and memory function. After the consumption of chungkookjang, daidzein is partially converted into equol by some gut microbiota, and isoflavone aglycones are absorbed in the intestines [63,64]. Equol is produced from daidzein by gut microbiota, including *Lactobacillus intestinalis*, *sakei*, *casei*, and *graminis* in rodents and humans [63,65]. However, not all people have equol-producing bacteria in the gut, and Asian women regularly consuming soybeans in their meals are more likely to have the bacteria [63]. Isoflavonoid aglycones, genistein, daidzein, and equol, are permeable to the blood–brain barrier, and they act directly to reduce neuroinflammation by suppressing TLR and NF-kB signaling, which leads to the release of proinflammatory cytokines and brain insulin resistance. The subsequent attenuation of inflammation suppressed tau phosphorylation in animal studies (Figure 2) [27,35,42]. Genistein acts as an inhibitor of γ-secretase [66]. The administration of fermented soybeans, including tempeh, has been shown to inhibit γ-secretase, thereby preventing an increase in the amyloid-β deposition of the brain in both in vitro and in vivo studies [67,68]. Thus, chungkookjang can decrease amyloid-β production by inhibiting γ-secretase. Isoflavone aglycones can directly reduce intracellular tau-related neurofibrillary tangles and extracellular amyloid-β deposition in the brain, especially the hippocampus, preventing and alleviating neuronal cell death in both in vitro and in vivo studies [20,65]. Dietary fiber and isoflavones in chungkookjang also modulate the gut microbiome to reduce neurotoxin production and elevate neuroprotection components such as butyrate, short-chain fatty acids (SCFA) [4,41]. These components produced by the gut microbiota may also alter systemic inflammation and insulin resistance to influence neuronal cell survival and death by the gut–microbiome–liver–brain axis. As a result, chungkookjang intake can protect against and alleviate Alzheimer’s symptoms, such as dementia in humans.

The extracellular deposition of amyloid-β and tau hyperphosphorylation in cells induces amyloid-β plaques and neurofibrillary tangles. The deposition of neurofibrillary tangles occurs when phosphorylated tau accumulates in the hippocampus in old IRS2-deficient mice [69]. The IRS2 gene expression is promoted by cAMP responding-element binding protein (CREB) in the pancreatic β-cells, liver, and brain of laboratory animals [70]. CREB itself acts as a critical factor for brain cell survival to suppress Alzheimer’s disease. The cAMP production activates protein kinase A (PKA) and CREB, and CREB binds to nuclear and mitochondrial DNA to promote neuronal survival genes, including BDNF and CNTF that protect against Alzheimer’s symptoms such as dementia [71]. CREB enhances neuronal cell survival by elevating the expression of BDNF and CNTF [5]. The high-dosage treatment of chungkookjang fermented with *B. amyloliquefaciens* SRCM 100730 and SRCM 100731 increased differentiated neuronal cell survival after infusion with amyloid-β (25–35) compared to the placebo control in an animal study [5]. BDNF and CNTF, indices of neuronal cell proliferation, were higher in cells treated with chungkookjang fermented with *B. amyloliquefaciens* SRCM 100730 and SRCM 100731 than in the placebo-control. The treatment with chungkookjang fermented with *B. amyloliquefaciens* also reduced SRCM73Tau mRNA expression more than that of the placebo control, and the reduction was a similar level as that in the normal control in an in vitro study [5]. Furthermore, chungkookjang intake enhanced the ability to secrete NGF and to modulate NGF receptor signaling in neuronal PC12 cells and the Tg2576 mouse model, which have the characteristics of Alzheimer’s disease. Chungkookjang intake in Tg2576 mice restored NGF secretion to that of the normal control [15]. In gerbil-induced cerebral ischemia, traditionally made chungkookjang and *B. licheniformis* inoculated chungkookjang (1–2 g/kg BW/day) prevented and alleviated neuronal cell death in the hippocampal CA1 region and neurological symptoms [35]. Chungkookjang intake reduces the expression of proinflammatory cytokines (TNF-α and IL-1β) in the hippocampus. Therefore, chungkookjang intake (about 15–30 g in human equivalents) prevents neuronal cell death, resulting in the prevention of memory impairment.

## 6. Improvement of the Gut–Liver–Brain Axis by Chungkookjang

### 6.1. Chungkookajng Effects on Gut Microbiota and SCFA as Synbiotics

The gut microbiota composition is highly variable among individuals. Its heterogeneity has an association with both intrinsic factors (genetics, genders, and age) and extrinsic factors (diet, herbs, polyphenols, age, antibiotics, lifestyle, and disease status) [72]. With aging, the diversity and amount of gut microbiota decrease. Many host diseases with aging are related to the composition of intestinal microbiota. However, the efficacies of gut microbiota modulations remain controversial. The intake of probiotics, prebiotics, and synbiotics contributes to the alteration of gut microbiota to reduce inflammation and insulin resistance, contributing to improved gastrointestinal and systemic health. Chungkookjang acts as a synbiotic to modulate gut microbiota [4]. In humans and animals, the gut microbiota is involved in gastrointestinal functions and other health-related functions, including energy, glucose, amino acid, and bone metabolism, as well as brain function [73,74]. These functions have bidirectional communications through the gut microbiota–liver and brain axis: SCFA production, inflammation, and immune response, and endocrine regulation including enteric hormones are the modulators from the gut to the liver and brain whereas the hypothalamic–pituitary–adrenal (HPA) axis, autonomous nervous system, and neurotransmitters deliver signals from the brain to the peripherals including the gut microbiota (Figure 2) [75]. These results suggest that brain function is involved in the gut microbiota–liver–brain axis in two-way communications.

SCFA, bile acids, trimethylamine-N-oxide, and immunoglobulin A produced by the gut microbiome act as metabolic modulators [76]. Gut microbiota directly activate the vagus nerve from the enteric nervous system to transmit the signals from the gut to the brain [77]. The activation of the HPA axis releases cortisol from the adrenal gland that influences gut microbiota composition and survival, which, in turn regulates the host immune response and other types of metabolism [77]. The enteric nervous system communicates with the central nervous system through the vagal nervous system, and gut microbiota are a modulator to control nutrient metabolism [78]. Gut microbiota modulate the secretion of gut hormones, including cholecystokinin, ghrelin, peptide YY, and glucagon-like peptide-1 (GLP-1). The gut hormones influence the vagal afferent pathway to modulate brain function and regulate intestinal metabolism [79]. These processes represent the gut microbiota–gut–brain bilateral communications that gut microbiota use to modulate brain function, including mood, emotions, neurodegeneration, and cognition (Figure 2) [78,80].

### 6.2. Chungkookajng Effect on the Gut-Microbiome-Gut-Liver-Brain Axis

A few studies have investigated gut microbiota changes that occur when consuming chungkookjang. Intake of chungkookjang fermented with *B. amyloliquefaciens* and *B. subtilis* increased *Bacillales*, *Lactobacillales*, and *Verrucomicrobiales* (*Akkermensia muciniphila*) and decreased *Enterobacteriales* in the cecum of type 2 diabetic rats [4]. Consumption of chungkookjang made with *B. amyloliquefaciens* SRCM 100730 and SRCM 100731 changed the amounts of *Bacteriodia* and *Clostridia* in ischemia-induced gerbils, similar to non-ischemic gerbils [42]. Furthermore, oral γ-PGA administration changed the relative abundance of *Lactobacillales* and *Clostridiales* in the large intestines of experimental animals [3]. The sizes of γ-PGA differently influence gut microbiota: 2000 and 2 kDa γ-PGA intake dramatically increased the relative abundance of *Lactobacillales* from 8% to 42% and 38%, respectively, whereas they decreased *Clostridiales* from 43% to 15% and 8%, respectively [3]. In particular, *L. intestinalis* survival increased from 0.9% to 23% in response to 2000 kDa γ-PGA and from 0.3% to 30% in response to 2 kDa γ-PGA in vitro. These results suggest that chungkookjang-enriched γ-PGA can promote the survival of *L. intestinalis* that is reported to efficiently synthesize equol from daidzein, especially from chungkookjang [63]. Therefore, oral administration of γ-PGA, especially the small size, can help modulate the gut microbiota as a prebiotic. 

Chungkookjang has insulinotropic activity, and it is associated with elevating the release of GLP-1 from L-cells to increase serum GLP-1 concentrations based on an animal study [14,81]. Chungkookjang intake also increased the production of SCFA and the integrity of intestinal tissues and decreased the production of proinflammatory cytokines in rodents [4]. The SCFA and cytokines enter to the bloodstream and are delivered into the liver and brain to modulate neuronal cell survival [20,82]. The modulation of the gut microbiome by chungkookjang induces changes that improved memory impairment in experimental animals [42]. Further studies need to research the mechanism of potentiating the axis by chungkookjang intake in humans. 

## 7. Conclusions

Soybeans contain various bioactive compounds that contribute to health benefits, and chungkookjang fermented with *B. amyloliquefaciens* has an acceptable flavor to most people. After their short-term fermentation with *Bacilli*, the fermented soybeans improve the utilization of the bioactive compounds such as increased isoflavone aglycones, smaller sizes of dietary fibers, γ-PGA, and peptides. Chungkookjang has better efficacy for type 2 diabetes and dementia than unfermented soybeans, shown mostly in experimental animals. Chungkookjang elevates the relative abundance of *Bacillales*, *Lactobacillales*, and *Verrucomicrobiales* (*Akkermensia muciniphila*), while it reduces the relative abundance of *Enterobacteriales* in the cecum. Thus, chungkookjang is considered a synbiotic. Chungkookjang made by fermenting the soybeans with *B. amyloliquefaciens* and *B. licheniformis* produces high concentrations of γ-PGA. The γ-PGA-rich chungkookjang has better efficacy for preventing and alleviating neuronal cell survival by improving brain insulin sensitivity and neuroinflammation and modulating the brain–liver–gut microbiota axis. Since a few studies on chungkookjang with respect to dementia have been conducted in human studies, the conclusions are extrapolated mainly from animal studies. Further research is needed to characterize the health-promoting activity of chungkookjang, including diabetic symptoms and memory impairment in human studies. 

## Figures and Tables

**Table 1:** The characteristics of chungkookjang fermented with different *Bacillus* species.

	*B. amyloliquefaciens*			*B. subtilis*	Soybeans
	SCGB 1	SRCM 100730	SRCM 100731	SCGB 574	
Number of bacteria (CFU/g)	2.2 × 10^10^ ± 2.6 × 10^9^	2.6 × 10^9^ ± 1.4 × 10^8^	4.7 × 10^9^ ± 2.8 × 10^8^	1.5 × 10^9^ ± 1.2 × 10^8^	5.0 × 10^3^ ± 2.6 × 10^2^
Number of *B. cereus* (CFU/g)	-	-	-	-	4.2 × 10^3^
γ-PGA (cm)	31 ± 0.86	27 ± 1.00	30 ± 0.57	55 ± 1.00	0 ± 0
Flavor	++	++	++	++	-
Protease activity (cm)	2.84 ± 0.04	1.94 ± 0.08	1.92 ± 0.12	2.29 ± 0.04	1.76 ± 0.01
Cellulase activity (cm)	2.08 ± 0.04	1.58 ± 0.04	1.44 ± 0.04	1.95 ± 0.07	1.78 ± 0.04
Amylase activity(cm)	2.84 ± 0.04	2.29 ± 0.05	2.42 ± 0.11	2.29 ± 0.04	1.99 ± 0.01
Thrombolytic activity (halo size, cm)	1.83 ± 0.06	3.85 ± 0.02	4.07 ± 0.14	1.97 ± 0.15	-

Values represent means ± standard deviations (*n* = 3). +, Detected; - Not detected. These results originated from previous research [24,25,26,27,28]. CFU, colony forming unit.

**Table 2:** Characteristics of *Bacillus* species isolated from traditionally made chungkookjang.

	*B. amyloliquefaciens*		*B. subtilis*	
	SCGB 1	SRCM 100730	SRCM 100731	SCGB 574
Survival rate in pH 2.0 (%)	0.45 ± 0.03	1.40 ± 0.11	6.91 ± 0.43	0.09 ± 0.11
Survival rate in pH 7.0 (%)	100	100	100	100
Survival rate in oxagall 0.3% (%)	19.83 ± 1.80	35.04 ± 1.74	20.18 ± 4.22	30.00 ± 3.17
Survival rate in oxagall 0.6% (%)	1.04 ± 0.11	57.97 ± 8.05	18.75 ± 4.09	3.29 ± 1.09
Tauro-deoxycholic acid hydrolysis activity (mm)	-	-	-	-
Glyco-deoxycholic acid hydrolysis activity (mm)	-	-	-	-
Antibacterial activity against *B. cereus* KCTC3624 (cm)	1.8 ± 0.3	1.4 ± 0.1	1.3 ± 0.1	1.8 ± 0.1
Antibacterial activity against *B. cereus* KCCM40935 (cm)	1.8 ± 0.17	1.4 ± 0.2	1.3 ± 0.11	2.0 ± 0.06
Antibacterial activity against *S. aureus* KCCM11593 (cm)	1.5 ± 0.1	1.1 ± 0.17	1 ± 0.15	No effect
Antibacterial activity against *S. aureus* KCCM41331 (cm)	1.0 ± 0.00	1.0 ± 0.1	1.0 ± 0.10	1.3 ± 0.1
Production of anti-bacterial components Surfactin	+++	+++	+++	+++
Iturin A	+	+++	+++	++
Bacillomycin D	+++	+++	+++	+++

Values represent means ± standard deviations (*n* = 3). +, Detected; - Not detected. These results originated from previous research [24,27,28,33].

## Data Availability

Not applicable.
